# The effect of transitioning to ICD-10-CM on acute injury surveillance of active duty service members

**DOI:** 10.1186/s40621-018-0162-y

**Published:** 2018-08-20

**Authors:** Matthew C. Inscore, Katherine R. Gonzales, Christopher P. Rennix, Bruce H. Jones

**Affiliations:** 1 Army Public Health Center, 5158 Black Hawk Rd, Aberdeen, MD 21010 USA; 2 Navy and Marine Corps Public Health Center, Naval Medical Center Portsmouth, 620 John Paul Jones Circle, Portsmouth, VA 23708 USA

**Keywords:** Barell matrix, Injury diagnosis matrix, ICD-9-CM, ICD-10-CM, Implementation, Surveillance, Military, Acute injury

## Abstract

**Background:**

Acute injuries are a burden on the Military Health System and degrade service members’ ability to train and deploy. Long-term injuries contribute to early attrition and increase disability costs. To properly quantify acute injuries and evaluate injury prevention programs, injuries must be accurately coded and documented. This analysis describes how the transition from International Classification of Diseases, Ninth Revision, Clinical Modification (ICD-9-CM) to the Tenth Revision (ICD-10-CM) impacted acute injury surveillance among active duty (AD) service members.

Twelve months of ICD-9-CM and ICD-10-CM coded ambulatory injury encounter records for Army, Navy, Air Force, and Marine Corps AD service members were analyzed to evaluate the effect of ICD-10-CM implementation on acute injury coding. Acute injuries coded with ICD-9-CM and categorized with the Barell matrix were compared to ICD-10-CM coded injuries classified by the proposed Injury Diagnosis Matrix (IDM). Both matrices categorize injuries by the nature of injury and into three levels of specificity for body region, although column and row headings are not identical.

**Results:**

Acute injury distribution between the two matrices was generally similar in the broader body region categories but diverged substantially at the most granular cell level. The proportion of Level 1 Spine and back Body Region diagnoses was higher in the Barell than in the IDM (6.8% and 2.3%, respectively). Unspecified Level 3 Lower extremity injuries were markedly lower in the IDM compared to the Barell (0.1% and 12.1%, respectively).

**Conclusions:**

This is the first large scale analysis evaluating the impacts of ICD-10-CM implementation on acute injury surveillance using ambulatory encounter data. Some injury diagnoses appeared to have shifted to a different chapter of the codebook. Also, it’s likely that the more detailed diagnostic descriptions and episode of care codes in ICD-10-CM discouraged re-coding of initial acute injury diagnoses.

The proposed IDM did not result in a major disruption of acute injury surveillance. However, many acute injury diagnosis codes cannot be aligned between ICD versions. Overall, the increased specificity of ICD-10-CM and use of the IDM may lead to more precise acute injury surveillance and tailored prevention programs, which may result in less chronic injury, reduced morbidity, and lower health-care costs.

## Background

Acute injuries are a burden on the Military Health System (MHS) and impact mission readiness by degrading service members’ (SMs’) ability to train and deploy. Long-term consequences of injury contribute to early attrition and increase disability costs for the Department of Defense (DoD). Across the armed services, acute injuries result annually in more than 700,000 outpatient visits costing billions of dollars (Inspector General of the United States Department of Defense, 2010).

The MHS served 1.35 million active duty (AD) SMs in calendar year (CY) 2016. Overall, more than nine million beneficiaries (i.e., SMs and their families) are eligible for care at military treatment facilities (MTFs), making the MHS one of the largest healthcare providers in the United States (Military health system, 2017). SM outpatient encounter data from around the world are captured and warehoused under uniform business rules. DoD epidemiologists conduct routine surveillance of acute injury and other medical conditions as part of the DoD’s commitment to safeguard the health of its SMs, monitor mission readiness, and evaluate the effectiveness of injury prevention programs.

The transition from International Classification of Diseases, Ninth Revision, Clinical Modification (ICD-9-CM) to the Tenth Revision (ICD-10-CM) on 01 October 2015 presented a challenge for acute injury surveillance. Differences between the two revisions are summarized elsewhere: the number of acute injury codes increased substantially in ICD-10-CM, resulting in greater granularity and rare one-to-one translations (Hedegaard et al., 2016). Also, the primary axis of classification in ICD-9-CM was the nature of injury, whereas the primary axis of classification in ICD-10-CM is the body region of injury (Hedegaard et al., 2016). The new ICD-10-CM revision was developed, in part, to improve the specificity of acute injury surveillance by providing information that was not captured under ICD-9-CM. Incident injuries coded with ICD-9-CM data were identified using other data (i.e., prior visit records) and were complicated by the lack of coding specificity (i.e., poor precision, no episode of care indicator, and no left-right body side indicator). In ICD-10-CM, the seventh character of the diagnosis code (A-C = initial; D-R = subsequent; S = sequela) shifts the burden of designating the episode of care to the provider.

Since its development, the Barell matrix has served as a basic, standardized surveillance tool for ICD-9-CM coded injury data nationwide. The Barell matrix has 12 nature of injury columns and 36 body region rows. Each ICD-9-CM code within the range of 800 to 995 is placed into a unique matrix cell. (Barell et al., 2002; National Center for Health Statistics, Centers for Disease Control and Prevention, 2010) The matrix rows and columns can be collapsed into broader groupings or expanded into more specific sites for additional detail. (Barell et al., 2002; National Center for Health Statistics, Centers for Disease Control and Prevention, 2010) The National Center for Health Statistics at the Centers for Disease Control and Prevention (CDC/NCHS) proposed an ICD-10-CM Injury Diagnosis Matrix (IDM) based on the Barell matrix and the ICD-10 Injury Mortality Diagnosis (IMD) matrix (Hedegaard et al., 2016; Fingerhut & Warner, 2006). Similar to the Barell matrix, the DoD modification of the IDM is a two dimensional matrix with 19 columns describing the nature of injury and body region categories organized into 36 rows (Hedegaard et al., 2016; Barell et al., 2002). Profound differences in organizational structure can be mitigated by cross-walking the two matrices, which are similar enough to facilitate categorical comparisons of injury distributions.

The IDM provides a framework for comparing categorized ICD-10-CM acute injury diagnoses to acute injury diagnoses classified by the ICD-9-CM Barell matrix. In many cases it is not possible to “cross-walk” Barell matrix cells directly to IDM cells. This is partly due to ICD-10-CM codebook containing 16 times the number of acute injury codes that ICD-9-CM had. More often than not, ICD-10-CM codes are more descriptive than ICD-9-CM codes, although sometimes the reverse is true.

In both the Barell and IDM matrices, body region categories are divided into three levels. Each successive level is progressively more detailed and nests within the previous level(s). A matrix cell is the most granular unit and one or more diagnosis codes may accrue to each cell within the matrices. A populated cell is treated as one injury regardless of how many diagnoses are assigned to the cell. For example, in both matrices, one or more finger fractures diagnosed in the same encounter would be assigned to a single cell intersecting “Fracture” and “Wrist, hand, and fingers,” although there may be fractures of multiple fingers. Multiple fractures could be differentiated in ICD-10-CM, whereas in ICD-9-CM, it would be unclear if the fracture was of a single bone or multiple bones of one or both hands. Regardless of the number of fractures that contributed to the cell, this example constitutes a single acute injury.

The purpose of this analysis is to determine the impact of ICD-10-CM implementation and the effects of utilizing the proposed CDC/NCHS IDM on acute injury surveillance among DoD AD SMs by comparing the classification and distribution of acute injuries using the Barell matrix and IDM.

## Methods

MHS administrative ambulatory encounter records for all Army, Navy, Air Force, and Marine Corps AD SMs with an acute injury diagnosis were abstracted from the Comprehensive Ambulatory/Professional Encounter Record (CAPER), for two one-year timeframes: 01 September 2014–31 August 2015 and 01 January – 31 December 2016 (DHSS Program Executive Office, 2018). The first timeframe captured acute injuries coded with ICD-9-CM diagnosis codes and the second timeframe captured injuries coded after the national implementation of the ICD-10-CM codebook. The ICD-10-CM timeframe was selected to exclude the first three months of ICD-10-CM implementation (i.e., October–December 2015) to allow sufficient time for coders to receive ICD-10-CM training and minimize the capture of early records that may have been incorrectly coded.

The DoD populations described here are rolling cohorts, but overall demographic characteristics of the populations (e.g., sex ratio, median age, age distribution, etc.) remain essentially constant. The populations within each timeframe were subject to the same fitness requirements (i.e., be fit enough to deploy) and had equal access to healthcare in the MHS. The DoD has been reducing the size of the military in recent years: sequential monthly snapshots demonstrate population counts declining during the timeframes. For reference, DoD population counts are reported for the month following each analytic timeframe (i.e., 30 September 2015 for the first timeframe and 31 January 2017 for the second timeframe) (Defense Manpower Data Center, n.d.).

This analysis utilized SAS software, Version 9.4 (SAS Institute, Inc., Cary, NC). An acute injury was defined as any ICD-9-CM code within the range of 800–995 in any primary and subsequent diagnostic fields (i.e., DX1-DX10). The Barell matrix was used to classify acute injuries by body region and nature of injury. An acute injury coded with ICD-10-CM was defined as any diagnostic code listed in the proposed ICD-10-CM IDM assignment table (all S and most T codes) developed by CDC/NCHS in any primary and subsequent diagnostic fields (i.e., DX1-DX10) (Hedegaard et al., 2016). The IDM was used to classify acute injuries by body region and nature of injury. In this analysis, four IDM column headings (i.e., “Effect of foreign body entering orifice,” “Other effects of external causes,” “Poisoning,” and “Toxic effects”) were grouped together and compared to the “Systemwide” category in the Barell matrix (Barell et al., 2002). The IDM Nature of Injury column titled “Muscles and tendons” is a new category and has no direct equivalent in the Barell matrix. Injuries classified as “Late effects” using ICD-9-CM codes (i.e., 905–909) or ICD-10-CM injury sequela codes (i.e., “S” in the seventh position) were not counted in the analysis.

Historic unpublished DoD acute injury surveillance experience has demonstrated that proportional distributions of injuries are comparable for similar cohorts across recent timeframes. In this analysis, the distributions of acute injuries categorized by the two matrices are compared by the three levels of body region categories and by nature of injury categories to evaluate acute injury diagnosing using different ICD versions.

Statistical evaluations of comparisons between the matrices were not conducted because they would have added little value to the discussion of the extent acute injury coding differs with ICD-10-CM compared to ICD-9-CM. There are approximately 16 times the number of acute injury ICD-10-CM diagnosis codes (i.e., Chapter 19: Injury, poisoning and certain other consequences of external causes) as there were ICD-9-CM acute injury codes (i.e., 800–995) in the Barell matrix. Whereas the authors believe statistical tests may be valid at the higher levels of aggregation of body regions and for most of the nature of injury levels, the large cohorts would have led to even slight differences being statistically significant. The intent here was to evaluate how well the IDM approximates the capture of acute injury compared to the Barell matrix.

This project was exempt from Institutional Review Board (IRB) review because the analysis was determined to be public heath practice and a non-research activity by the Navy and Marine Corps Public Health Center assistant research coordinator. In addition, the data were accessed retrospectively and the analysis did not involve observation of or interaction with SMs.

## Results

For the ICD-9-CM timeframe, the reported end-of-year DoD AD population was 1,313,940 and the reported end-of-year ICD-10-CM population was 1,292,519, a decline of 21,421 SMs, or 1.6% (Defense Manpower Data Center, n.d.). From 01 September 2014–31 August 2015, there were 661,027 acute injury diagnoses identified using the Barell matrix. The IDM identified 588,284 acute injury diagnoses during CY 2016. While the DoD AD population declined 1.6%, the decline in the number of acute injury diagnoses was approximately 10.8%.

The distributions of injury diagnoses in the Level 1 Body Region of Injury categories were similar between the Barell matrix and the IDM (Tables 1, 2). Level 1 “Extremities” injury encounters accounted for the majority of injuries at 69.1% and 74.2% in the Barell and IDM, respectively. The “Head and neck” category accounted for the second-highest proportion of injuries: 10.5% in the Barell matrix and 13.2% in the IDM. Injuries within the “Spine and back” category accounted for 6.8% in the Barell matrix, but only 2.3% in the IDM. The IDM “Torso” level categories were reorganized and direct comparisons between the matrices were not possible beyond Level 1 (Tables 1, 2). However, the proportions of injuries in the Level 1 “Torso” category compared favorably across the two matrices at 6.2% and 6.4% in the Barell and IDM, respectively. “Unclassifiable” injuries in the Barell matrix, including “Other and unspecified” and “Systemwide” injuries, accounted for nearly twice the number of equivalent injuries in the same IDM categories: 7.5% and 4.0%, respectively.

**Table 1:**
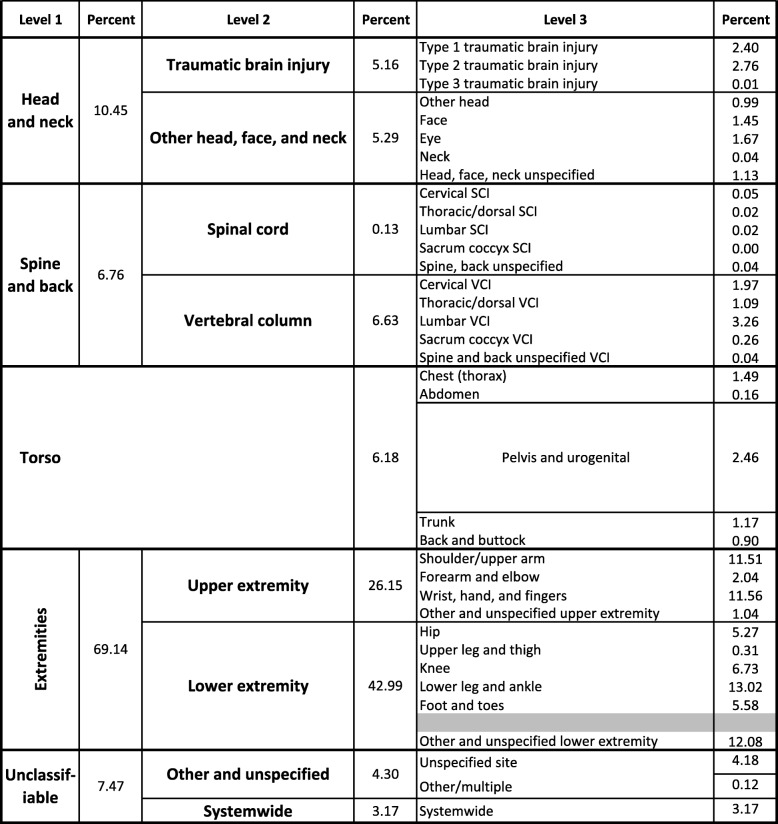
Acute Injuries among Military Personnel, by Body Region, Barell Matrix (ICD-9-CM), *N* = 661,027

Shaded rows intentionally left blank where the Injury Diagnosis matrix and Barell matrix do not directly align

**Table 2:**
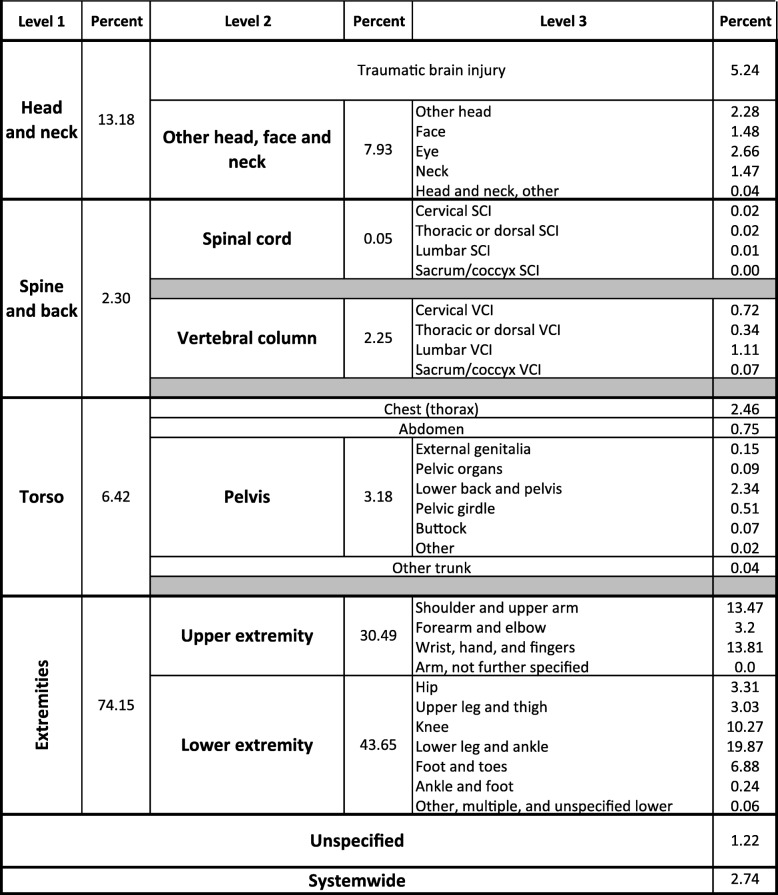
Acute Injuries among Military Personnel, by Body Region, Injury Diagnosis Matrix (ICD-10-CM), *N* = 588,284

Shaded rows intentionally left blank where the Barell Matrix and Injury Diagnosis Matrix do not directly align

Distributions of injuries in the Level 2 Body Region of Injury categories were similar between the two matrices (Tables 1, 2), although somewhat less so than for Level 1 categories. “Lower extremity” injuries accounted for the plurality of encounters: 43.0% in the Barell matrix and 43.7% in the IDM. “Upper extremity” injuries accounted for the second highest proportion of injuries: 26.2% in the Barell matrix compared to 30.5% in the IDM. Proportions of head injuries were the same for “Traumatic brain injury” (5.2%). The most notable divergence was the “Vertebral column” category, which accounted for 6.6% of acute injuries in the Barell matrix but only 2.3% in the IDM.

There were substantial deviations in the Level 3 Body Region of Injury categories between the matrices. Two of the largest disparities were seen in the Level 3 “Lower extremity” category, but when aggregated to Level 2 categories, the proportions of acute injuries attributable to “Lower extremity” deviated less than 1% between the matrices. “Lower leg and ankle” injuries accounted for 13.0% of acute injuries in the Barell matrix compared to 19.9% of acute injuries in the IDM. The most striking difference was in the Barell matrix “Other and unspecified lower extremity” category, which comprised 12.1% of injuries compared to the IDM “Other, multiple, and unspecified lower extremity” category at 0.1% of injuries. The six well-defined Level 3 categories in the IDM that cross-walk to a single Barell matrix Level 3 grouping (i.e., “Pelvis and urogenital”) may have been influenced by the change in the primary axis of classification to the body region in the IDM.

Injury distributions in the Nature of Injury categories were similar between the Barell matrix and the IDM (Tables 3, 4). “Sprains and strains” accounted for the majority of injuries in the Barell matrix (51.7%) compared to a plurality of injuries in the IDM (47.9%, including “Muscles and tendons”). The new “Muscles and tendons” category accounted for 3.7% of IDM injuries. “Fractures” accounted for 12.9% of injuries in the Barell matrix and 16.0% in the IDM. The first three columns of the Barell matrix (i.e., “Fracture,” “Dislocation,” and “Sprains and strains”) and the first four columns of the IDM (i.e., “Fracture,” “Dislocation,” “Sprains and strains,” and the new “Muscles and tendons”) yielded similar proportions of injuries in the aggregate: 69.3% and 67.0%, respectively. There was good agreement across the remaining Nature of Injury columns between the matrices.

**Table 3:**
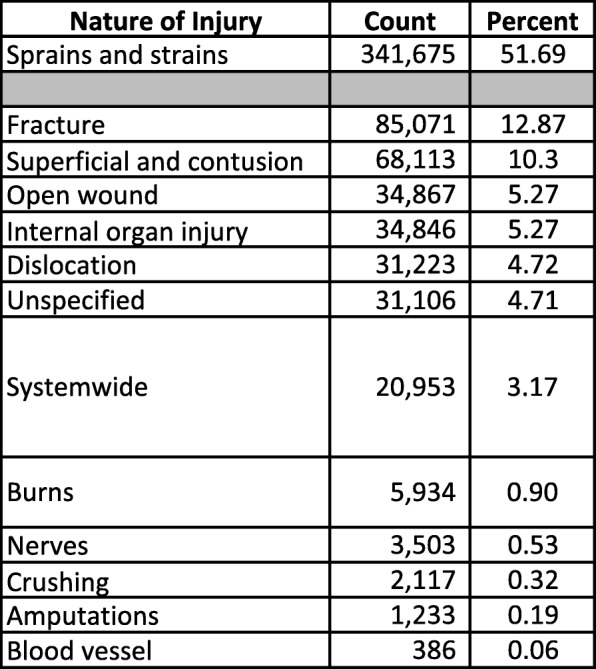
Acute Injuries among Military Personnel, by Nature of Injury, Barell Matrix (ICD-9-CM), *N* = 661,027

Shaded rows intentionally left blank where the Barell Matrix and Injury Diagnosis Matrix do not directly align

**Table 4:**
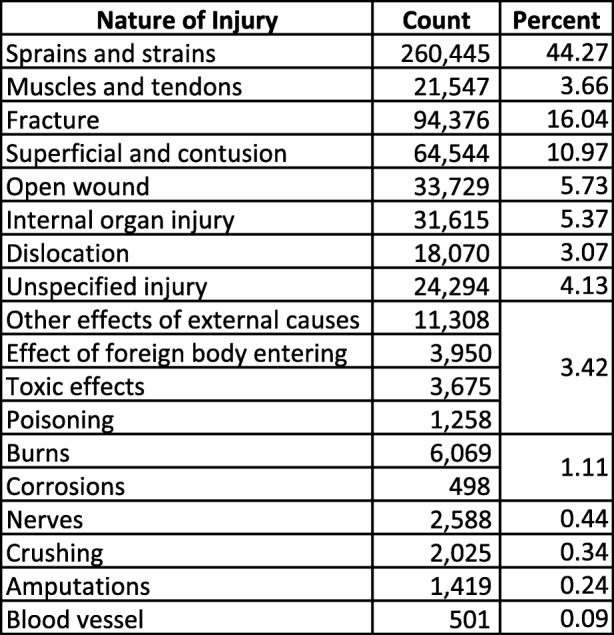
Acute Injuries among Military Personnel, by Nature of Injury, Injury Diagnosis Matrix (ICD-10-CM), N=588,284

## Discussion

To the authors’ knowledge, this is the first large scale analysis evaluating the impacts of ICD-10-CM implementation on acute injury surveillance using ambulatory encounter data. This comparison has a number of strengths. Unlike civilian healthcare systems, the MHS operates under a uniform set of business rules, including the TRICARE healthcare plan, which is managed by the Defense Health Agency (DHA) which is the predominant entity managing medical claims for AD personnel. This eliminates several confounders, such as unequal access to healthcare, income inequality, place of residence, etc. In addition, the large population size, generally healthy population, and personal identifiers create a powerful and unprecedented analytical tool.

Within the large, demographically stable AD cohort, the proportional distributions of acute injuries by body regions and nature of injury are predictable from year to year. The small variation in annual injury rates (not reported) does not seem to adequately account for the substantial drop in acute injury diagnosis counts between the two ICD versions, only one year apart. The discrepancy is likely attributable to the greatly expanded lexicon of detailed acute injury codes in ICD-10-CM. The new IDM Nature of Injury Category “Muscles and tendons” accounted for 3.7% of acute injuries. Most of those injury assignments would likely have been coded as “Sprains and strains” in the Barell matrix. However, differences in the distribution of acute injuries between the two matrices are not solely the result of how acute injuries are defined within each matrix. The collections of acute injury codes in each matrix do not set the limits for diagnostic options as there are thousands of potential diagnoses outside the matrices (especially in ICD-10-CM) that may affect the relative proportions of acute injury distributions within the matrices.

The ICD-9-CM/ICD-10-CM coding guidelines define late effects and sequela using identical language: “A late effect [ICD-9-CM]/sequela [ICD-10-CM] is the residual effect (condition produced) after the acute phase of an illness or injury has terminated.” (Centers for Medicare and Medicaid Services and the National Center for Health Statistics, 2011; Centers for Medicare and Medicaid Services and the National Center for Health Statistics, 2017) The guidelines also employ identical language in the coding of residual effects: “The code for the acute phase of an illness or injury that led to the late effect/sequela is never used with a code for the late effect.” (Centers for Medicare and Medicaid Services and the National Center for Health Statistics, 2011; Centers for Medicare and Medicaid Services and the National Center for Health Statistics, 2017) The logic is to preclude re-coding of acute injuries in follow-up encounters that could not be discerned from incident injuries. The issue is ostensibly addressed in ICD-10-CM by the seventh position that denotes the episode of care, which allows an antecedent injury to be documented without ambiguity. If an ICD-10-CM injury code indicates a sequela, there should be no mistaking the code for an incident injury. It is not possible with ICD-9-CM coding to confidently discern sequela or episode of care of a specific acute injury.

Although laterality (i.e., left or right side of the body) was not captured in ICD-9-CM, it is captured in ICD-10-CM. The proposed IDM does not categorize acute injuries by laterality. Analyses revealed that laterality coding was often inconsistent for a single acute injury, shifting among left, right, and unspecified over a sequential series of encounters. It seems likely that this reflects careless coding rather than true, bilateral injuries. Like the Barell matrix, the IDM aggregates related groups of acute injury diagnoses into single cells for surveillance. Except for a small number of traumatic injuries (e.g., amputations), categorization by laterality may offer limited utility for surveillance. In cases of multi-trauma, laterality may be determined after first identifying the general body locations and nature of injuries.

Prior to 01 October 2015, it appears that MHS providers routinely used acute injury diagnoses to record injuries that were antecedent to the condition currently being treated. DoD epidemiologists have seen an apparent increase in the coding of musculoskeletal conditions, which are not acute injuries. It seems likely that the more detailed diagnoses available in ICD-10-CM describing arthropathies, auto-inflammatory syndromes, inflammatory conditions, and other joint disorders outside of chapter 19: “Injury, poisoning and certain other consequences of external causes” adjust for what would have been improperly re-coded acute injuries under ICD-9-CM, thus reducing the counts of acute injury diagnoses.

There were far fewer “Spine and back” (Body Level 1) injuries identified in the IDM than in the Barell matrix. Vertebral column injuries (Body Level 2) also comprised a far lower percentage of total injuries in the IDM compared to the Barell matrix. These injuries may have migrated to a different chapter of the codebook, such as chapter 13: “Diseases of the musculoskeletal system and connective tissue.” Again, it seems likely that the more detailed diagnosis descriptions and episode of care codes in ICD-10-CM may discourage re-coding of initial acute injury diagnoses.

Proportions for “Lower extremity” injuries were the same between the two matrices (43.0% in the Barell and 43.6% in the IDM). The smaller percentage of IDM unspecified lower extremity injuries appears to be explained by the greater specificity of ICD-10-CM coding. The majority of injuries formerly categorized as “Other and unspecified lower extremity” in the Barell matrix may have shifted to the more refined “Lower leg and ankle” (Level 3) or even the more specific Level 3 “Knee” category in the IDM.

The Body Level 1 “Unclassifiable” category in the Barell matrix accounted for a far greater proportion of acute injury diagnoses than the comparable category(ies) in the IDM. This suggests that the far more descriptive, and far more numerous, ICD-10-CM codes made it possible for providers to avoid some of the general diagnoses that were unspecified as to the precise location of the injury and even the exact nature of injury. For this analysis, the proposed IDM “Multiple Body Regions” was dropped and the categories within the Barell matrix “Unclassifiable” body level were aligned with the IDM categories “Unspecified” and “Systemwide.” This was the second largest Body Level 1 disparity next to “Spine and back.” A review of Body Level 3 proportions reveals nearly three times the proportion of injuries in the Barell matrix “Spine and back” as in the IDM. This is not surprising as reductions in the proportions of nonspecific diagnoses are seen elsewhere in the aligned matrices, most strikingly in the Body Level 3 comparison of the Barell “Other and unspecified lower extremity” category and the IDM “Other, multiple, and unspecified lower extremity” category.

Most DoD providers in the ambulatory setting code their own injury encounters using an application built into the electronic medical record (EMR) software. Such systems make it easy for a busy provider to code the first menu option that is “close enough.” Prior to ICD-10-CM implementation, providers were given training on the new ICD version, although the thoroughness of training and length of training may have varied by MTF and medical specialty. Inconsistent training and local work cultures may have affected coding of acute injury diagnoses. Previous research on injury cause coding documented coding deficiencies for a five year period following the transition from a familiar coding system to an unfamiliar one (i.e., ICD-9 to ICD-10) (Nilson et al., 2015). Ambulatory encounter data used in this analysis revealed numerous examples of improper coding that did not follow ICD-10-CM guidelines, such as subsequent diagnoses with no antecedent initial diagnosis, thousands of sequelae codes in the primary diagnosis position, inconsistent laterality coding, etc. These, and other coding errors, could be addressed by adjustments to the EMR software and improved training for medical providers.

### Limitations

The DoD AD population differs from the United States employed civilian population: service members are mostly male (84%) and young (75% are under the age of 35 compared to the civilian employed population where less than 40% are under the age of 35) (United States Department of Labor, 2017). The DoD AD cohorts compared were from different one year timeframes that were nearly adjacent. Given the consistent requirements of military service, the populations were strikingly similar in terms of age, sex, service affiliation, duty station locations, occupations and access to care. Therefore, the differences in the cohorts were likely negligible relative to how they might have affected behaviors in seeking medical care for acute injuries. These findings may not be generalizable to acute injuries treated in the civilian healthcare sector. Although MTFs report reimbursable medical care under a uniform set of business rules, those rules may not be the same as those that govern reimbursable care in civilian healthcare systems. Therefore, the distribution(s) of acute injury diagnoses may be different between military and civilian sectors even after controlling for age and sex distributions, access to care, the healthy worker effect, etc. The data analyzed excluded care provided in deployed settings or any medical care an AD SM may have received from a civilian provider.

The intent was not to evaluate the accuracy of diagnosing using ICD-10-CM and no chart review was conducted to evaluate the appropriateness of providers’ coding choices. The gold standard for determining the definitive explanation for the 10.8% decline in acute injury diagnoses coded with ICD-10-CM might be to have providers code all encounters with both ICD-9-CM and ICD-10-CM among a single cohort. Although possible on a small scale, such an undertaking was beyond the resources available for this investigation.

The analysis utilized a single year of ICD-10-CM data. This comparison was necessary as DoD epidemiologists are tasked with ongoing acute injury surveillance. Further study and evaluation using multiple years of acute injury data coded with ICD-10-CM are needed to elucidate the full impact of ICD-10-CM implementation on acute injury surveillance.

## Conclusions

The CDC/NCHS-proposed IDM effectively captures acute injuries coded in ICD-10-CM similar to those captured in the ICD-9-CM Barell matrix. Proportional distributions of injuries by “Nature of Injury” categories are comparable between the matrices. “Body Region” agreement of injury distributions between the matrices is generally very good at the broadest Level 1 and Level 2 categories. Discrepancies among injury distributions at Level 3 are largely attributable to the improved specificity of ICD-10-CM coding and often represent a shift away from “unspecified” categories under ICD-9-CM. Providers do not always follow episode of care coding guidelines, but the 10.8% drop in injury diagnoses seen in the IDM compared to the Barell may represent a more accurate capture of acute injuries in the DoD MHS. The results of this comparative analysis illustrate that MHS providers may be coding acute injuries less frequently using the far more descriptive ICD-10-CM version than they did under ICD-9-CM. This suggests that the transition to ICD-10-CM may have improved the accuracy and precision of acute injury coding in the outpatient setting. If acute injury coding practices in the civilian health care sector are comparable to those in the MHS, data derived from the IDM may substantially inform researchers and healthcare managers when facing decisions about resource allocation and interventions.

## Abbreviations

AD: Active duty
CAPER: Comprehensive Ambulatory/Professional Encounter Record
CDC/NCHS: National Center for Health Statistics at the Centers for Disease Control and Prevention
CY: Calendar year
DHA: Defense Health Agency
DoD: Department of Defense
EMR: Electronic medical record
ICD-10-CM: International Classification of Diseases, Tenth Revision, Clinical Modification
ICD-9-CM: International Classification of Diseases, Ninth Revision, Clinical Modification
IDM: Injury Diagnosis Matrix
IMD: Injury Mortality Diagnosis
IRB: Institutional Review Board
MHS: Military Health System
MTF: Military treatment facility
SM: Service member
